# Peritoneal Dialysis—Moving from Current Status to the Future

**DOI:** 10.1155/2013/530713

**Published:** 2013-10-24

**Authors:** Kook-Hwan Oh, Yong-Lim Kim, Wai-Kei Lo, Peter J. Margetts

**Affiliations:** ^1^Nephrology Division, Department of Internal Medicine, Seoul National University Hospital, 101 Daehakro, Chongno Gu, Seoul 110-744, Republic of Korea; ^2^Nephrology Division, Kyungpook National University Hospital, Daegu, Republic of Korea; ^3^Division of Nephrology, University of Hong Kong, Hong Kong; ^4^Department of Medicine, McMaster University, Hamilton, ON, Canada

Peritoneal dialysis (PD) has been widely used as one of the major renal replacement therapies (RRT) for end-stage renal disease (ESRD) patients. Although PD therapy has witnessed remarkable technical advances, and the patient's survival early after starting PD is comparable, or superior, to that of hemodialysis, it is currently faced with many challenges. Long-term PD is associated with progressive loss of UF capacity, resulting in increased cardiovascular morbidity or ultimate discontinuation of PD. This is related to inflammation, new vessel formation (angiogenesis), and fibrotic thickening of the peritoneal membrane (PM). Bioincompatible PD fluid, along with peritonitis, is the major contributor to the PM change. In order to establish PD as a more general and longstanding renal replacement therapy, clinicians and scientists need to investigate the pivotal issues such as adequate volume control and long-term preservation of the peritoneal membrane and the prevention from devastating conditions such as encapsulating peritoneal sclerosis.

In this special issue, we have compiled elegant reviews and clinical studies with a special interest in the above-mentioned “*Contemporary issues of PD therapy*.” This special issue deals with many of the current issues such as PD catheter implantation by nephrologists, clinical benefits of the newer biocompatible PD fluid, peritonitis, and how to cope with low ultrafiltration volume with bimodal and twice-daily icodextrin use and several others. It is the hope of our editorial committee that this special issue will contribute to an improved patient care and serve as a stimulus to seek a new knowledge in renal replacement therapy.

*Kook-Hwan Oh* *Kook-Hwan Oh* *Yong-Lim Kim* *Yong-Lim Kim* *Wai-Kei Lo* *Wai-Kei Lo* *Peter J. Margetts* *Peter J. Margetts*

